# Targeted Deep Sequencing Uncovers Cryptic KIT Mutations in KIT/PDGFRA/SDH/RAS-P Wild-Type GIST

**DOI:** 10.3389/fonc.2020.00504

**Published:** 2020-04-22

**Authors:** Annalisa Astolfi, Valentina Indio, Margherita Nannini, Maristella Saponara, Angela Schipani, Antonio De Leo, Annalisa Altimari, Bruno Vincenzi, Danila Comandini, Giovanni Grignani, Paola Secchiero, Milena Urbini, Maria Abbondanza Pantaleo

**Affiliations:** ^1^Department of Morphology, Surgery & Experimental Medicine, University of Ferrara, Ferrara, Italy; ^2^“Giorgio Prodi” Cancer Research Center, University of Bologna, Bologna, Italy; ^3^Department of Experimental, Diagnostic and Specialty Medicine, S. Orsola-Malpighi Hospital, University of Bologna, Bologna, Italy; ^4^Medical Oncology Unit, S.Orsola-Malpighi University Hospital, Bologna, Italy; ^5^Laboratory of Oncologic Molecular Pathology, S. Orsola-Malpighi Hospital, Bologna, Italy; ^6^Department of Medical Oncology, University Campus Bio-Medico, Rome, Italy; ^7^Medical Oncology 1, Policlinico San Martino, University of Genova, Genova, Italy; ^8^Sarcoma Unit, Candiolo Cancer Institute - FPO, IRCCS, Candiolo, Italy

**Keywords:** gastrointestinal stromal tumor, GIST, KIT, quadruple-WT, deep sequencing

## Abstract

**Background:** Gastrointestinal stromal tumors (GIST) are known to carry oncogenic KIT or PDGFRA mutations, or less commonly SDH or NF1 gene inactivation, with very rare cases harboring mutant BRAF or RAS alleles. Approximately 10% of GISTs are devoid of any of such mutations and are characterized by very limited therapeutic opportunities and poor response to standard treatments.

**Methods:** Twenty-six sporadic KIT/PDGFRA/SDH/RAS-pathway wild type GIST were profiled for the molecular status of genes frequently altered in GIST by a targeted next generation sequencing (NGS) approach. Molecular findings were validated by alternative amplicon-based targeted sequencing, immunohistochemistry, gene expression profiling and Sanger sequencing.

**Results:** Three patients harboring NF1 inactivating mutations were identified and excluded from further analysis. Intriguingly, five patients carried cryptic KIT alterations, mainly represented by low-allele-fraction mutations (12–16% allele ratio). These mutations were confirmed by another targeted NGS approaches and supported by CD117 immuno-staining, gene expression profiling, Sanger sequencing, with peak signals at the level of background noise, and by the patients' clinical course assessment.

**Conclusion:** This study indicates that ~20% patients diagnosed with a KIT/PDGFRA/SDH/RAS-pathway wild-type GIST are *bona-fide* carriers of pathogenic KIT mutations, thus expected to be eligible for and responsive to the various therapeutic lines of TK-inhibitors in use for KIT/PDGFRA-mutant GIST. The centralization for a second level molecular analysis of GIST samples diagnosed as wild-type for KIT and PDGFRA is once again strongly recommended.

## Introduction

Gastrointestinal stromal tumors (GISTs) are rare mesenchymal tumors arising in the gastrointestinal tract (1). Nearly 85% of GISTs are characterized by mutually exclusive activating mutations in KIT or PDGFRA receptors (2, 3), that lead to constitutive ligand-independent activation of receptor signaling and account for their sensitivity to tyrosine kinase inhibitors (TKI) (4, 5). TKIs are the standard therapeutic approach for patients with unresectable tumor, ensuring a significant improvement in the clinical outcome of patients with advanced disease. Approximately 10–15% of all sporadic GIST cases are devoid of mutations in either gene, and have always been classified as KIT/PDGFRA wild-type GIST (6). This very heterogeneous category includes around 20–40% of cases that are succinate dehydrogenase complex (SDH)-deficient GIST, due to germline and/or somatic loss-of-function mutations in any of the four SDH subunits (A, B, C, or D) (7–9). Another subgroup of KIT/PDGFRA wild-type GIST with intact SDH complex, collectively defined as RAS-pathway (RAS-P)-mutant GIST, includes patients that either carry inactivating mutations in NF1 gene, often signaling an unrecognized NF1 syndromic condition (10, 11), or activating mutations in BRAF or more rarely a RAS gene (12, 13). Hence there is only half of the KIT/PDGFRA WT GIST that are recognized as either SDH-deficient or BRAF/RAS/NF1 mutated, but still the other half remains orphan of a driver oncogenic event and possibly of a specific target for therapy. Up to now, only private or hardly recurrent alterations have been identified in this GIST subgroup, such as ETV6-NTRK3, FGFR1, or FGF4 alterations, MAX, MEN1 (14–17), and still no conclusive result has been found on the actionable mutations for this subset of patients. Conversely, many studies point to a common gene expression profile (15, 18), despite the heterogeneity of the genetic analysis, suggesting that some shared signaling pathway should be evoked by different genetic alterations.

Hence, in this study we sought to investigate in depth, by a targeted NGS approach, the genetic status of the so-called KIT/PDGFRA/SDH/RAS-P wild-type GIST, to uncover putative alterations in frequently mutated genes that could be missed by conventional molecular diagnostic approaches.

## Methods

### Patient Series

The series consisted of archival FFPE tissues from 26 GIST specimens negative for KIT/PDGFRA/BRAF/NRAS/KRAS with intact SDH complex, that are designated here as quadruple-WT for clarity. GIST diagnosis was done by expert pathologists based on morphology and CD117 expression. The study was approved by the local Institutional Ethical Committee and informed consent was provided by all living patients.

KIT, PDGFRA, BRAF, KRAS, and NRAS mutational status was assessed by Sanger sequencing both by the local diagnostic service and replicated and confirmed by our referral Molecular Diagnostic Unit. In KIT/PDGFRA/BRAF/KRAS/NRAS-negative cases, SDH deficiency was assessed by IHC for SDHB, followed by Sanger sequencing of the four SDH subunits.

### Targeted Deep Sequencing

Areas with more than 90% of tumor cells were selected by an expert pathologist and dissected for nucleic acid extraction. DNA was extracted using QiAmp DNA micro Kit (QIAGEN) and quantified using picogreen dsDNA assay (Life Technologies). TruSeq Custom Amplicon (TSCA) low input sequencing panel, covering the entire coding region of NF1, SDHA, SDHB, SDHC, SDHD, and selected exons of KIT (exons 8, 9, 11, 13, 14, 17, 18), PDGFRA (exons 12,14,18), BRAF (exons 11 and 15), NRAS (exons 2 and 3), and KRAS (exons 2, 3 and 4), was designed with Design Studio software (Illumina). All KIT and PDGFRA exons target of primary or secondary mutations indicated in the most recent guidelines on GIST molecular diagnostics were included in this panel (19, 20). BRAF, NRAS, and KRAS recurrent hotspot mutations were covered. Since the DNA was extracted from Formalin-Fixed Paraffin-Embedded (FFPE) specimens, we employed a dual-strand TruSeq Custom Amplicon (TSCA) approach, that is able to discriminate reads produced from positive and negative strands of DNA to exclude artifacts derived from tissue fixation. The average amplicon length was of 175 bp. Thirty nanograms of DNA extracted from 26 FFPE GIST samples were used for library synthesis following TSCA Low-Input Dual Strand kit (Illumina) guidelines. Briefly, for each region of interest, two custom probes were hybridized and elongated copying target DNA. The two elongation products were then ligated and amplificated adding Illumina adaptes and sequencing primers Illumina adapters and sequencing primers.

Libraries were then quantified using Quant-IT Picogreen dsDNA reagent (Thermo Fisher Scientific), normalized to 4 nM and pooled. Ten picomolar (pM) of pooled libraries were sequenced on a Micro V2 flowcell on Miseq platform (Illumina) at 150 bp read length in paired-end mode, reaching an average depth of 295X.

To confirm the presence of low-allele-fraction mutations in the KIT gene, a complementary targeted sequencing approach was also employed, based on deep sequencing of PCR amplicons of target KIT exons. DNA library preparation was performed with Nextera-XT DNA library prep kit (Illumina) following manufacturer's recommendations. Amplicons of the corresponding regions were prepared by PCR reaction with Phusion Hot Start II DNA Polymerase (Thermo Fisher Scientific) using custom-designed primers for each exon (primer sequence available upon request). Deep sequencing was performed on the MiSeq System (Illumina) at 150 bp read length in paired-end mode, reaching an average depth of coverage of 9900X.

### Bioinformatics Analysis

Amplicon sequencing was analyzed using a customized pipeline. For Illumina workflow, demultiplexing was performed with Miseq Reporter 2.6 (Illumina) and the paired-end reads were aligned on GRCh38 human reference genome. BAMClipper tool was adopted to perform soft-clipping in order to remove amplicon primers from alignment. Single nucleotide variants were called with SNVMix2 tool while insertions and deletions were called with the HaplotypeCaller function of GATK3 adopting a combination of optional parameters suited to detect variation with low-allele-frequency small mapping quality (–minimum-mapping-quality 10; –max-alternate-alleles 1; –sample-ploidy 8; –max-reads-per-alignment-start 1,000). All variants were annotated with Annovar and filtered according to Exac minor allele frequencies, 0.1 altered allele fraction and at least a total depth of coverage of 20X and 5X of the altered allele. Moreover, variants detected in only one strand were considered as FFPE artifacts.

### RNA- Sequencing

Total RNA was extracted using RecoverAll Total Nucleic Acid Isolation Kit (Thermo Fisher Scientific) and used for cDNA library synthesis using TruSeq RNA Exome kit (Illumina) according to manufacturer's instructions. Single cDNA libraries were pooled and hybridized to a set of probes covering 45 Mb of coding exonic regions. Paired-end libraries were then sequenced at 2 × 80 bp on a NextSeq500 instrument (Illumina), producing an average of 51.1 × 10^6^ reads per sample. After FASTQ generation and trimming of low-quality bases and sequencing adapters, paired-end reads were aligned with the TopHat/BowTie pipeline and gene expression was quantified with the package HTSeq-count and normalized as count per million (CPM) using the R-bioconductor pakage edgeR. The set of genes differentially expressed (*p*-value <10^−3^) between KIT-mutant and quadruple-WT GIST was obtained with the R-bioconductor package *limma* (lmfit an eBayes functions). The list of selected genes was used to perform hierarchical clustering of the low-allele-fraction KIT-mutant sample with the R-bioconductor package pheatmap (clustering distance: correlation; clustering method: complete).

### PCR, qPCR, and Sanger Sequencing

KIT exon 9 and 11 were re-sequenced on FFPE tumor specimens using the Sanger sequencing method on ABI 3730 Genetic Analyzer (Applied Biosystems, Monza, Italy). Primer pairs, designed with Primer Express 3.0 Software (Applied Biosystems), were specific to amplify exons and part of the flanking intronic regions. PCR products were sequenced on both strands using the Big Dye Terminator v1.1 Cycle Sequencing kit (Applied Biosystems) on a ABI 3730 Genetic Analyzer (Applied Biosystems).

FGF4 copy number status was measured on ABI Prism 7900HT platform (Applied Biosystems) using FAM-labeled TaqMan Copy Number Assays (Thermo Fisher Scientific) targeting FGF4 (Hs02374436_cn) and XXRA1 (Hs03782780_cn), located in chromosome bands 11q13.3 and 11q13.4, respectively. TaqMan RNaseP Control Reagent (VIC-labeled) was used as internal reference control. Estimation of FGF4 copy number was done using DDCt method in comparison with XRRA1 and with a normal diploid sample as a calibrator.

### Immunohistochemistry

Immunohistochemical analysis for CD117/c-Kit was performed on 3 μm paraffin-embedded tumor sections using monoclonal pre-diluted anti-CD117 clone YR145 (Ventana Medical Systems, USA) on Ventana Benchmark Ultra platform. Antigen Retrieval was performed in UltraCC1 Tris-HCl buffer pH 8.2–8.5 at 95°C for 24–48 min, and the immunologic reaction was visualized with the OptiView DAB Detection Kit (Ventana, USA).

## Results

The series consisted of 26 GIST specimens selected as negative for KIT/PDGFRA/BRAF/NRAS/KRAS mutations and with intact SDH complex, whose molecular characterization was performed by Sanger sequencing and immunohistochemistry. These samples were analyzed by means of a custom NGS amplicon approach targeting key genes frequently altered in GIST (KIT, PDGFRA, BRAF, NRAS, KRAS, SDHA, SDHB, SDHC, SDHD, and NF1), reaching an average depth of coverage of 295X. Overall, three samples carrying NF1 loss-of-function mutations were identified, and therefore excluded from further analyses (Table 1). These tumors were found to carry clearly pathogenic mutations, either truncations (p.Q519X and Q959X in GIST_406 and GIST_251 respectively) or frameshift mutations (p.R1241fs in GIST_203).

**Table 1:** List of pathogenic mutations identified by targeted deep sequencing.

**ID**	**Gene**	**Mutation (exon, cDNA, protein)**	**Type of mutation**	**Depth of coverage**	**Mutant allele frequency**
GIST_406	NF1	Exon 22, c.C2875T, p.Q959X	Stop Gain	123X	47%
GIST_251	NF1	Exon 14, c.C1555T, p.Q519X	Stop Gain	213X	37%
GIST_203	NF1	Exon 28, c.3721delC, p.R1241fs	Frameshift deletion	403X	93%
GIST_260	KIT	Exon 11, c.T1657C, p.W557R	Missense	396X (18379X)	**14%** (23%)
GIST_241	KIT	Exon 9, c.1502_1503insTGCCTA, p.S501delinsSAY	In-frame insertion	391X (5763X)	**12%** (9%)
GIST_307	KIT	Exon 11, c.1723_1724insAACTTCCTTATG, p.Q575delinsQLPYE	In-frame insertion	468X	**16%**
GIST_218	KIT	Exon 11, c.1726insC;1726_1764dup, p.L576_R588dup	In-frame insertion	274X	**12%**
GIST_169	KIT	Exon 11, c. 1648_1672del, p.550_558del	In-frame deletion	100X	49%

*The table lists the depth of coverage and the mutant allele frequency of the TSCA target sequencing assay. In brackets there are the same values for the KIT exon 9 and exon 11 Nextera-XT PCR amplicon assay*.

*Bold values indicate the samples with low-allele-fraction mutations*.

More interestingly, among the 23 remaining cases, five patients (22%) were unexpectedly found to carry pathogenic alterations in the KIT gene (Table 1). One case (GIST_169) showed a large deletion of 32 nucleotides (c. 1648_1672del) overlapping the intron-exon boundary upstream of exon 11 (Table 1). This deletion removes the 5′-splice site, and introduces a new donor splice site, coupled to the deletion of the first nine amino acids from the mature protein. Likely this event is not routinely detected by molecular diagnostic procedures since the deletion removes seven nucleotides from the flanking intronic sequence, where usually sequencing primers are located. The deletion was confirmed through Sanger sequencing using appropriate primers (Supplementary Figure 1).

**Figure 1:**
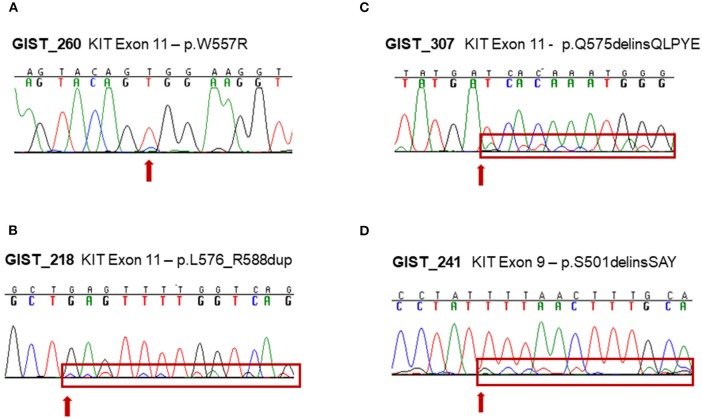
Experimental validation of low-allele-fraction mutations in GIST samples by Sanger sequencing. **(A)** KIT exon 11 c.T1657C mutation in GIST_260. **(B)** KIT exon 11 c.1726insC;1726_1764dup mutation in GIST_218. **(C)** KIT exon 11 c.1723_1724insAACTTCCTTATG in-frame insertion in GIST_307. **(D)** KIT exon 9 c.1502_1503insTGCCTA in-frame insertion in GIST_241.

The other four samples were instead carriers of a low-allele-fraction KIT mutation, with a detected altered allele frequency of 12–16% (Table 1). Three mutations affected KIT exon 11: a missense p.W557R mutation in GIST_260 and two non-frameshift alterations (p.L576_R588dup and p.Q575delinsQLPYE) in GIST_218 and GIST_307. The other mutation detected was p.S501delinsSAY involving exon 9 in GIST_241. These four events were clearly below the detection limit of conventional Sanger sequencing, even if the mutations were noticeable in the electropherogram at the level of background signal modifications (Figures 1A–D). The presence of these low-allele-fraction mutations was confirmed also through an independent NGS assay, based on deep sequencing of PCR amplicons targeting only KIT exon 9 and 11. This approach, that reached a minimum coverage of 9900X per sample, yielded very similar KIT-mutant allelic frequencies in GIST_241 and GIST_260, with a ratio of 9 and 23%, respectively (Table 1, *in brackets*). Besides, since this targeted sequencing approach uses different primers pairs to amplify KIT exons, we can rule out that the low ratio of the mutant allele is due to an artificial allelic dropout during DNA amplification.

**Figure 2:**
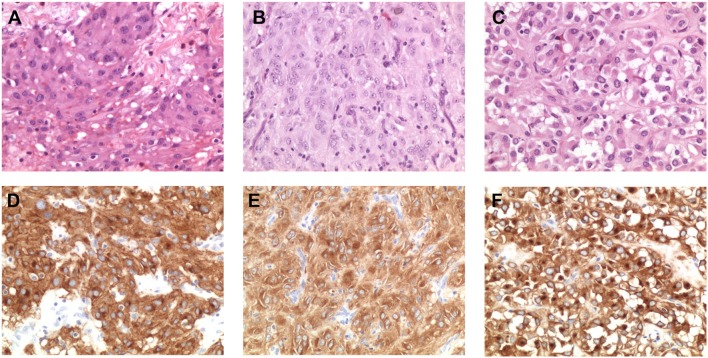
Morphological and immunohistochemical analysis of low-allele-fraction KIT-mutant GIST. **(A)** GIST_260 appears as an epithelioid gastrointestinal stromal tumor comprising polymorphous cells arranged in nests and sheets with eosinophilic cytoplasm and peripherally placed nuclei, with mild nuclear atypia and no necrosis. The mitotic rate is 3/50 high power fields (HPF). (Hematoxylin and eosin stain: original magnification x 200). **(B)** GIST_241 is composed by spindle cell arranged in short fascicles and whorls, with pale eosinophilic fibrillary cytoplasm, ovoid nuclei, and ill-defined cell borders. The mitotic rate is 20/50 HPF. (Hematoxylin and eosin stain: original magnification x 200). **(C)** GIST_307 appears as an epithelioid gastrointestinal stromal tumor, composed by round cells with clear to eosinophilic cytoplasm arranged in sheets and nests, with low-grade nuclear atypia, mild nuclear pleomorphism and indistinct nucleoli. The mitotic rate is 2/50 HPF. (Hematoxylin and eosin stain: original magnification x 200). **(D)** CD117/KIT staining in GIST_260 reveals a strong positivity with membrane, cytoplasmic and “dot-like” Golgi staining (original magnification x 200). **(E)** KIT staining in GIST_241 shows a diffuse cytoplasmic staining (original magnification x 200). **(F)** Immunohistochemical analysis of CD117 expression in GIST_307 shows a strong and diffuse cytoplasmic and paranuclear Golgi-like staining (original magnification x 200).

To ensure that the tumor area was correctly isolated and dissected prior to nucleic acid extraction, we performed histopathological revision of the FFPE blocks of three of the four cases harboring low-allele-fraction KIT mutations (GIST_260, GIST_241 and GIST_307). An expert pathologist selected again the tumor area containing more than 90% of tumor cells (Figures 2A–C) and DNA was extracted and sequenced by Sanger method. The presence of the low-allele-fraction mutation was confirmed in both cases, with profiles comparable to the ones resulting from the previous nucleic acid extraction, confirming that these alterations were indeed low frequency alleles (*data not shown*). CD117 immunostaining was strongly positive in all patients, as expected since almost all GISTs show CD117 expression. Interestingly, GIST_260 and GIST_307 additionally showed a combined membranous, cytoplasmic, and paranuclear Golgi-like positivity, suggestive of a diffuse alteration of KIT expression in the tumor mass (Figures 2D–F). It is noteworthy that Golgi-like staining, that is significantly more frequent in KIT-mutant than in WT-GIST (21), was detected in two low-allele-fraction mutant samples.

**Figure 3:**
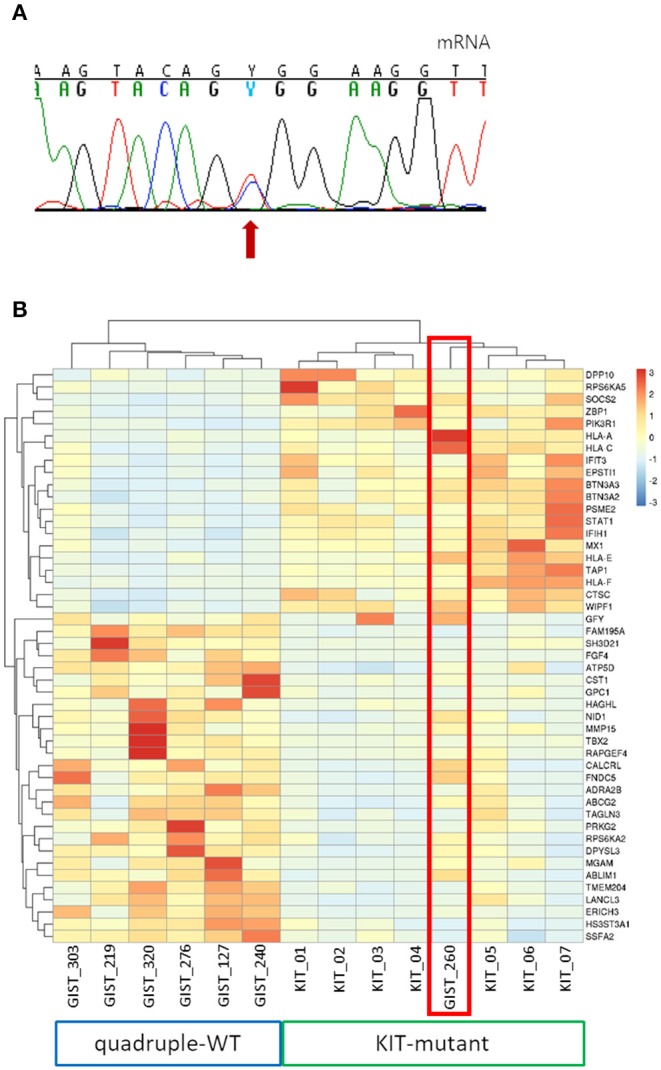
Analysis of KIT-specific gene expression in GIST_260. **(A)** Mutant allele expression in GIST_260 mRNA. Despite the low-allele-fraction in DNA, the mutant allele is highly expressed. **(B)** Hierarchical clustering of the top genes differentially expressed between the seven KIT-mutant cases and the six quadruple-WT (*p* < 10^−3^). GIST_260 clusters with KIT-mutant samples.

In one of the four cases (GIST_260) whole transcriptome sequencing and targeted KIT mRNA sequencing was performed, revealing a high expression of the mutant allele, despite the low allelic fraction at the DNA level (Figure 3A). Furthermore, this sample clustered with KIT-mutant samples with respect to the genes differentially expressed between quadruple-WT and KIT-mutant GIST (Figure 3B). Of relevance, GIST_260 did not express FGF4, that is selectively upregulated in quadruple-WT cases and is not expressed in KIT-mutant GIST (16). FGF4 copy number status was also measured in the low-allele-fraction samples, confirming the absence of FGF4 gain (Supplementary Figure 2), that we showed as a feature of quadruple-WT GIST (16).

Lastly, the clinical course of the four patients carrying low-allelic-fraction KIT mutations was analyzed, showing that one of the four patients (GIST_307) developed peritoneal metastasis during the disease course (Table 2). The patient was treated with imatinib for 3 years and the survival from the time of metastatic relapse lasted for 40.5 months, an interval that is comparable to the median survival time of KIT/PDGFRA-mutant metastatic patients (56.6 months) and definitely higher than that of quadruple-WT GIST (25.2 months) (Supplementary Figure 3), thus reinforcing the relevance of low-allele-fraction KIT mutations in driving TKI-response in GIST.

**Table 2:** Clinical and demographic data of the low-allele-fraction KIT-mutant patients.

**ID**	**Age range**	**Site**	**Size (cm)**	**Mitotic count**	**Risk classification**	**Metastasis**	Status^*^
GIST_260	51–55	Jejunum	11	2	High	No	NED
GIST_241	71–75	Ileum	11	>5	High	No	NA
GIST_307	61–65	Ileum	8.5	<5	High	Peritoneum	DOD
GIST_218	56–60	Ileum	7	4	Intermediate	No	AWD

* *Patients' status at last follow up: NED, no evidence of disease; DOD, died of disease; AWD, alive with disease; NA, not available*.

Collectively these data indicate that roughly one out of five patients diagnosed with a KIT/PDGFRA/SDH/RAS-P wild-type GIST is a *bona-fide* carrier of pathogenic KIT mutation, thus expected to be eligible for and responsive to the various therapeutic lines of TK-inhibitors approved for KIT/PDGFRA-mutant GIST.

## Discussion

In this study we analyzed a series of 26 GIST negative for KIT/PDGFRA/BRAF/NRAS/KRAS mutations and with intact SDH complex, analyzed in two different Diagnostic Centers (local and referral), identifying three NF1-mutated samples, in agreement with a previous study showing a relevant frequency of NF1 mutations in quadruple-negative GIST cases (11). Quite unexpectedly, we identified five cases carrying pathogenic KIT mutations, which means that a fraction of more than one out of five apparently quadruple-WT GIST actually turns out to be KIT–mutant. Thus, our results demonstrate that a significant fraction of GIST patients actually affected by a KIT–mutant tumor are missed by the state-of-the-art molecular diagnostic protocols due to the limits of the standard techniques in use. Therefore, in practice, the fraction of patients affected by a KIT/PDGFRA WT GIST should be considered lower than currently expected. As a matter of fact, large deletions involving exon-flanking regions can be missed through allelic dropout while low-allele-fraction mutations are routinely overlooked by conventional Sanger sequencing due to the inherent detection limit of the approach. Indeed, a low frequency KIT mutation was already reported in a previous study made with an amplicon sequencing approach, where an exon 11 V561D was described at 9% allele frequency in a GIST specimen (22). More importantly, a recent large scale genomic study of more than 5,000 tumor samples definitely showed that a low-allele-fraction mutations in cancer samples is a surprisingly frequent condition, with a routine detection of hotspot mutations in actionable genes such as EGFR, KRAS, PIK3CA and BRAF with an allele fraction below 10% in about 20% of clinical samples (23). Besides, in this study the authors also show that cases with low mutant allele frequency respond to TKI target therapy at the same level of cases with high allele frequency mutations, indirectly proving that low frequency mutations are biologically meaningful and clinically actionable (23). Likewise, we showed that GIST_307, carrying a low-allele-fraction KIT mutation, exhibited a long-term response to imatinib, with an overall survival of more than 3 years after metastatic spread of the disease.

The reasons for the presence of low-allele-fraction mutations in cancer samples are various and complex, ranging from intra-tumor spatial heterogeneity to FFPE-induced degradation and chemical modification of DNA, that can impact on target amplification efficiency and reliability (24). Indeed, tumor heterogeneity is supposed to play a major role in low-allele-fraction mutations, an issue that can be highly relevant for necrotic tumors, since a recent study revealed an allele ratio of the same driver mutation in different samplings of the same GIST specimen from 10% to up to 60% (25). All these factors, coupled with the low sensitivity of Sanger sequencing, are supposed to play a role in the occurrence of low-allele-fraction mutations, that are supposed anyway to behave as clinically actionable alterations (23).

These findings underline once again the importance to refer patients with KIT/PDGFRA WT GIST to high-volume molecular diagnostic centers as already also suggested by the recent clinical guidelines (26), in which the implementation of appropriate next-generation-sequencing panels could be used to address the few cases with cryptic KIT mutations.

Quadruple-WT GIST represent an undefined and heterogeneous category of tumors (15, 27), that inevitably poorly respond to standard treatments, represented by TKI, due to the lack of the target oncogenic alteration. The detection of a significant fraction of this subgroup as carrier of actionable KIT mutations not only advocates the routine implementation of next generation sequencing approaches in the current molecular diagnostic protocols, but also opens new and effective therapeutic strategies for these patients, that are actually devoid of active pharmacological opportunities. As a matter of fact, our findings suggest that, in the metastatic setting, patients with a diagnosis of a KIT/PDGFRA WT GIST, except for those with known therapeutic molecular targets (involving BRAF or NTRK or FGFR), should always be treated with imatinib because the event of a cryptic KIT mutation may occur. In these cases, the predictive role of baseline and 1-month FDG-PET could assist the physicians in the early evaluation of imatinib response in clinical practice (28).

In conclusion, this analysis demonstrates that a significant proportion of quadruple–WT GIST patients are actually carrying pathologically relevant low-allele-fraction KIT mutations, that would benefit from TKI treatments both in the adjuvant and metastatic setting and that should be readily identified at the early diagnostic stage though implementation of appropriate next-generation-sequencing panels and addressing to national hub diagnostic centers. These results warrant further investigations to confirm in a wider series that in 20% of KIT/PDGFRA/SDH/RAS-pathway wild-type GIST it is possible to find cryptic KIT alterations.

## Data Availability Statement

The datasets generated for this study can be found in the Sequence Read Archive (SRA) (https://www.ncbi.nlm.nih.gov/sra) (PRJNA602810).

## Ethics Statement

The studies involving human participants were reviewed and approved by Ethical committee of Azienda Ospedaliero-Universitaria Policlinico S. Orsola-Malpighi. The patients/participants provided their written informed consent to participate in this study.

## Author Contributions

MP, MU, and AAs: study concept and design. AAs, MU, AS, MN, MS, AD, AAl, and PS: acquisition of data. BV, DC, GG, and MP: patients' clinical supervision. VI: data analysis. AAs, MU, and MP: drafting of the manuscript. AAs, MU, VI, MN, MS, and MP: study supervision. All authors read and approved the final manuscript.

## Conflict of Interest

The authors declare that this study received funding from Petra S.r.l., Fondazione Isabella Seràgnoli Onlus and Fondazione Mafalda Righi Onlus. The funders were not involved in the study design, collection, analysis, interpretation of data, the writing of this article, or the decision to submit it for publication.
