# Supplementary material for: Targeted Deep Sequencing Uncovers Cryptic KIT Mutations in KIT/PDGFRA/SDH/RAS-P Wild-Type GIST
Source: Front Oncol. 2020 Apr 22;10:504. doi: 10.3389/fonc.2020.00504 (PMC7188756; doi:10.3389/fonc.2020.00504)
Supplement: Supplementary file 1 [file Presentation_1.pptx]

## Slide 1
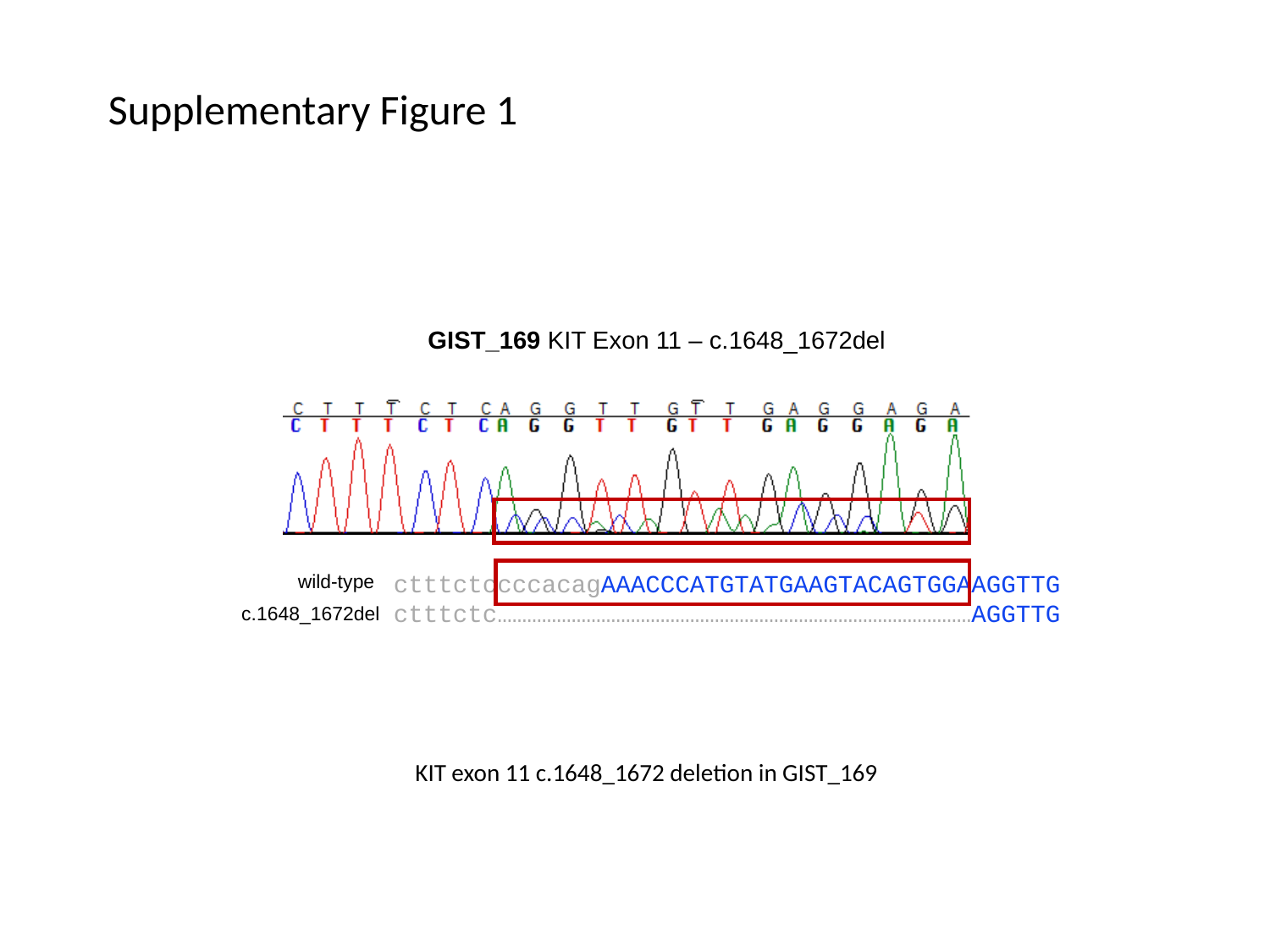

Supplementary Figure 1
GIST_169 KIT Exon 11 – c.1648_1672del
ctttctccccacagAAACCCATGTATGAAGTACAGTGGAAGGTTG
ctttctc……………………………………………………………………………………AGGTTG
wild-type
c.1648_1672del
KIT exon 11 c.1648_1672 deletion in GIST_169

## Slide 2
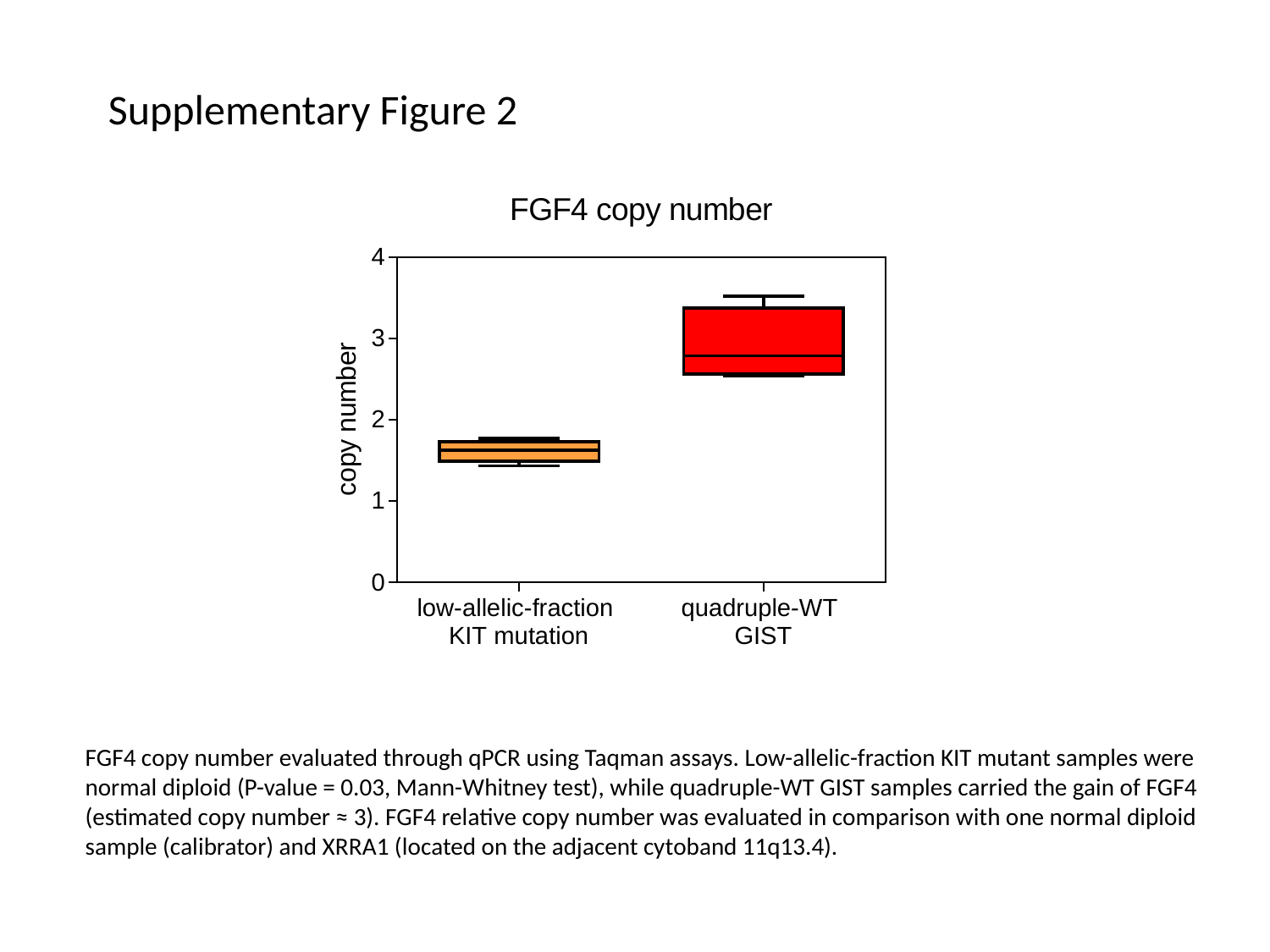

Supplementary Figure 2
FGF4 copy number evaluated through qPCR using Taqman assays. Low-allelic-fraction KIT mutant samples were normal diploid (P-value = 0.03, Mann-Whitney test), while quadruple-WT GIST samples carried the gain of FGF4 (estimated copy number ≈ 3). FGF4 relative copy number was evaluated in comparison with one normal diploid sample (calibrator) and XRRA1 (located on the adjacent cytoband 11q13.4).

## Slide 3
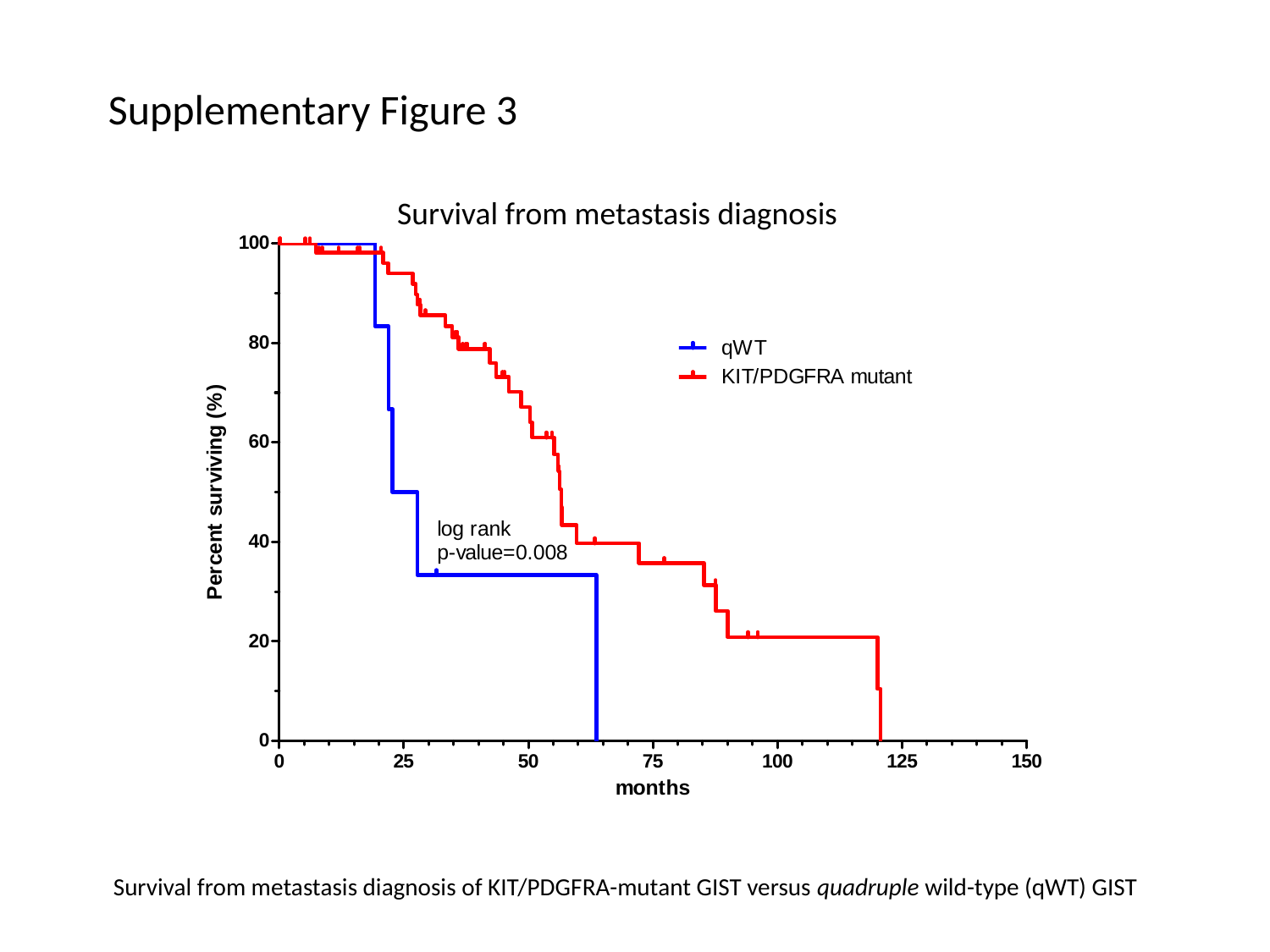

Supplementary Figure 3
Survival from metastasis diagnosis
Survival from metastasis diagnosis of KIT/PDGFRA-mutant GIST versus quadruple wild-type (qWT) GIST
